# Vegetarianism

**Published:** 1862-11

**Authors:** 


### VEGETARIANISM.

Those who are impatient of the bold repetition of old and refuted follies; who are angry with the apostles of an error who for ever preach as truths the rankest nonsense, the most worn-out fallacies, and the most childish gossip,—will have read with a feeble stir of indignation and contempt the reports of a recent meeting of the "Vegetarian Society." It is difficult to conceive a more condensed series of absurdities, errors, and misstatements than has been produced by the clever reduction into bare sentences of the opinions broached, for the purpose of abridged report in the public journals. The absurdities of the proceedings are most cleverly presented. Vegetarianism has not flourished because, while men are engaged in studying the means of destroying each other without compunction, it is not likely that they will systematically consider the life of cows, sheep and, such low animals. It is true that such Brahminical reverence does not greatly influence our feeding at present; and if any religious arguments are to be drawn from the Bible, they assuredly are far from discountenancing the consumption of animal food, since lists of lawful food are there given: while the observation of the ordained manner of living of that large class of creatures which are destined to prey upon the vegetable-feeding animals affords a natural-history precedent. The boldness with which all the convincing series of facts is ignored is characteristic of this most absurd of systems. One speaker is described as saying, "Animals fed upon vegetable products; and all the nutriment combined in the flesh of these animals was derived from that source. Why, then, should men obtain nutriment at second hand, alloyed by the impurities of the animals through which it had passed?" As though all animals feed upon vegetable food? The notion of describing butcher's meat as second-hand cabbage and potatoes alloyed with animal impurities, is an exquisite specimen of physiology for the people. If this speaker will observe the grinding teeth, the triple stomachs, the chewing propensities, the complicated structure and enormous length of the digestive intestinal canal in vegetable-feeding animals, and the single stomach, cutting teeth, and simple intestinal apparatus of man, he will appreciate the anatomical reasons which have determined the flesh-eating propensities of our race. Perhaps there is not any one of our habits or natural instincts of which the cause is so clearly apparent in the elements of our structure, of which the religious approbation is more deeply founded, or of which the utility is more logically evident. Of the personal experiences related at this meeting we need not say anything; leaving the illness of the gentleman who suffered "jaundice as the consequence of chill after a plentiful meal off cheese-curds" to the special interpretation of the pathologists of his school.

But why is this school of nonsense called "Vegetarianism" at all? The name is a mockery. The diet which these enthusiasts adopt is more animal than that of half the meat-eaters; and it has been calculated by no less competent an authority than Dr. Carpenter, that in adopting the rules in a well-known "vegetarian" diet-book the quantity of eggs, milk, and butter consumed would give half an ounce more animal matter daily in the diet than in that of ordinary families. These gentlemen have, then, a great objection to second-hand grass alloyed with animal impurity in the form of flesh, but not in the form of milk and eggs. It must be a source of wonder that, spite of the inexhaustible folly and onesideness of mankind, a delusion so transparent as this should exist even in small proportions.

It is hardly consolatory, although in some sense satisfactory, to know that a heresy exists in the opposite direction, and that in a work published not long since in Liverpool the author announces his conviction that the human race is essentially intended to be carnivorous; and incidentally mentions in the course of his argument that he is on the lookout for a lady of similar convictions, who will unite her fortunes with his, and adopt his diet, with the view of bringing up a family of carnivorians. Horribly suggestive is the notion of the possible development of such a family, if the Darwinian hypothesis be well grounded. What might be the specific changes earnest from such an application of the principles of election to the origin of species it is hard to say; but this is a rival absurdity to "vegetarianism" which may amusingly serve to antagonize it.

